# Endoscopic Characteristics of the Healing Process of Ulcerative Colitis

**DOI:** 10.1155/DTE.5.37

**Published:** 1998

**Authors:** Hideyuki Kishi

**Affiliations:** Third Department of Internal Medicine Toho University School of Medicine 2-17-6 Ohashi, Meguro-ku Tokyo 153 Japan

## Abstract

This study compared the histologic characteristics of ulcerative colitis with findings on conventional colonoscopy and on magnification and dye application for 70 sites that underwent biopsy. The primary objective was to study the correspondence between histologic findings and endoscopic findings with respect to glandular restructuring and the resolution of inflammation from the active to the remission phase of ulcerative colitis. Widened grooves, as assessed by the endoscopic staining technique and magnified observation, most closely correlated with histologic evidence of resolution of inflammation, and vascular markings and color tone of the mucosa on general colonoscopy most closely correlated with histologic evidence of glandular restructuring, such as glandular maturity. Magnifying endoscopy after dye application, in addition to conventional endoscopy, is therefore considered essential in the evaluation of ulcerative colitis during the resolving phase.


*Diagnostic and Therapeutic Endoscopy*, Vol. 5, pp. 37–48
Reprints available directly from the publisher
Photocopying permitted by license only

© 1998 OPA (Overseas Publishers Association) N.V.
Published by license under
the Harwood Academic Publishers imprint,
part of The Gordon and Breach Publishing Group.
Printed in Singapore.

### Endoscopic Characteristics of the Healing Process of Ulcerative Colitis

HIDEYUKI KISHI

*Third Department of Internal Medicine, Toho University School of Medicine, 2-17-6 Ohashi, Meguro-ku, Tokyo 153, Japan*

*(Received 6 May 1997; In final form 30 March 1998)*

**This study compared the histologic characteristics of ulcerative colitis with findings on conventional colonoscopy and on magnification and dye application for 70 sites that underwent biopsy. The primary objective was to study the correspondence between histologic findings and endoscopic findings with respect to glandular restructuring and the resolution of inflammation from the active to the remission phase of ulcerative colitis. Widened grooves, as assessed by the endoscopic staining technique and magnified observation, most closely correlated with histologic evidence of resolution of inflammation, and vascular markings and color tone of the mucosa on general colonoscopy most closely correlated with histologic evidence of glandular restructuring, such as glandular maturity. Magnifying endoscopy after dye application, in addition to conventional endoscopy, is therefore considered essential in the evaluation of ulcerative colitis during the resolving phase.**

*Keywords:* Widened grooves, Magnifying endoscopy after dye application, Resolving phase of ulcerative colitis

### 1 INTRODUCTION

Ulcerative colitis has an active phase, characterized by endoscopic evidence of ulceration, erosion, bleeding and edema, and a resolving phase, characterized by the resolution of these findings and, occasionally, the appearance of inflammatory polyps. Histologically, the process from the active phase to the resolving phase involves the gradual resolution of inflammation. The number of neutrocytes decreases and chronic inflammatory cell infiltration resolves; restructuring of the tubular epithelium may also occur. The relation between these histologic changes and endoscopic manifestations of the disease remains to be established. A clearer understanding of this relation may contribute to the establishment of improved criteria for the endoscopic classification of ulcerative colitis and for the evaluation of therapeutic response. We therefore compared the histologic characteristics of ulcerative colitis with findings on conventional colonoscopy and on magnification and dye application. The primary objective was to study the correspondence between histologic findings and endoscopic findings with respect to glandular restructuring and the resolution of inflammation from the active to the resolving phase of ulcerative colitis.

### 2 MATERIALS AND METHODS

Between 1991 and 1995, a total of 52 patients with ulcerative colitis underwent endoscopic examination of the lower gastrointestinal tract at our institution. For this study, we enrolled 10 patients (9 men and 1 woman; mean age 37.5 ± 15.0 years) with ulcerative colitis of the entire colon that was in remission clinically. Four patients had initial episodes, and 6 had remissions after exacerbations of disease. All patients gave their informed consent to participate in the study and undergo endoscopy and biopsy. The patients were pre-treated with 2000 ml of polyethylene glycol-electrolyte lavage solution [1] and examined endoscopically. An endoscope manufactured by the Olympus Co., Ltd. (model CF200Z; Tokyo, Japan) was used for examination of the colon. The scope was inserted into the cecum or into the oral side of the lesion border. Generally, observations were made during withdrawal of the scope.

During the active phase, sites confirmed to be lesions on endoscopy or on barium enema radiography were examined by conventional endoscopy, and vascular markings and reddening were evaluated. Edema and friability were not included in the final analysis because their evaluations were affected by the endoscopic procedure used for examination. Vascular markings were graded as absent (–), partly indistinct in some areas (±), and distinct (+). Reddening was evaluated as marked (+), spotty or faint (±), or none (–) (Table I).

A 0.5% methylene blue solution was then applied under direct vision with the use of a cleaning tube (Olympus Co., Ltd.; model, PW-6P). The presence or absence of defects of the mucosa was evaluated on the basis of the accumulation pattern of the dye immediately after application. Ulceration or erosion was considered positive (+) if the diameter of the dyed area was 5 mm or more, marginal (±) if the diameter was less than 5 mm, and negative (–) if there was no evidence of dye accumulation (Table I).

TABLE I Grading of colonoscopic findings

| | |
|---|---|
| Conventional observations | |
| a. Vascular markings | – :absent |
| | ± :partly indistinct in some areas |
| | + :distinct |
| b. Reddening | + :marked |
| | ± :spotty or faint |
| | – :none |
| Surface dyeing | |
| c. Erosion or ulceration | + :5 mm or more |
| | ± :less than 5 mm |
| | – :none |
| d. Width of innominate grooves between mucosal areas | + :widened |
| | ± :mixture of widened and normal |
| | – :normal |

The sites applied with dye were magnified 20–30 times, and the width of innominate grooves between mucosal areas was evaluated. The width of the innominate grooves was evaluated as positive (+) if there was consistent widening of the grooves (Fig. 1), marginal (±) if there was a combination of widened grooves and grooves similar in width to those of the normal mucosa (Fig. 2), and negative (–) if the grooves were similar in width to those of the normal mucosa (Fig. 3) (Table I). After evaluation, each case was documented photographically, and a specimen was taken from the same site by targeted biopsy. A total of 70 biopsy specimens were taken from the following sites: Cecum, 2 specimens; ascending colon, 5 specimens; hepatic flexure, 5 specimens; transverse colon, 10 specimens; splenic flexure, 5 specimens; descending colon, 14 specimens; sigmoid colon, 18 specimens; rectum, 11 specimens (Table II).

The biopsy specimens were fixed in a solution of 10% formalin, embedded in paraffin, thinly sliced into sections (thickness, 4 μm), and stained with hematoxylin and eosin. Histologically, chronic inflammatory cell infiltration was graded as severe (++), mild (+), or normal (±). Since the

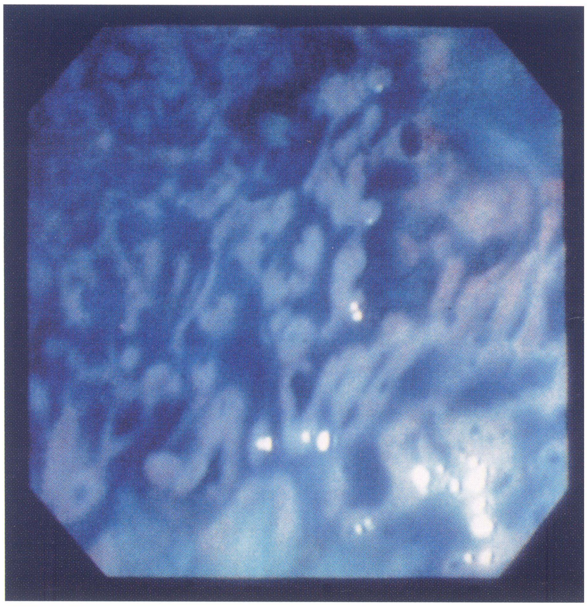

FIGURE 1 Colonoscopic findings after dyeing. Widened grooves can be seen.

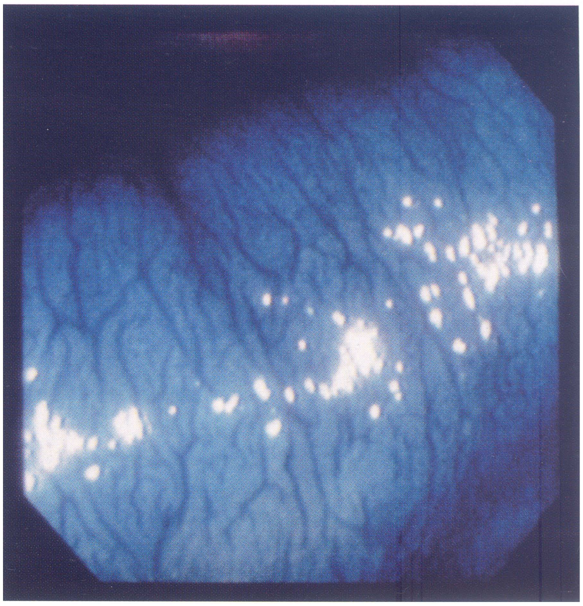

FIGURE 3 Colonoscopic findings after dyeing. Normal grooves.

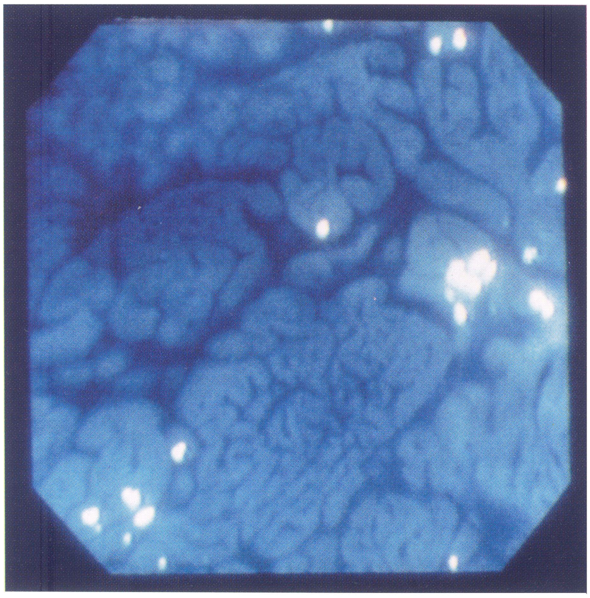

FIGURE 2 Colonoscopic findings after dyeing. A mixture of widened and normal grooves can be seen.

TABLE II Site of biopsy and number of specimens

| Site | No. of specimens |
|---|---|
| Cecum | 2 |
| Ascending colon | 5 |
| Hepatic flexure | 5 |
| Transverse colon | 10 |
| Splenic flexure | 5 |
| Descending colon | 14 |
| Sigmoid colon | 18 |
| Rectum | 11 |
| | Total 70 |

resolution of inflammation is reported to be associated with inflammatory cell infiltration of the lamina propria [11], cases with severe (++) chronic inflammatory cell infiltration were classified as active stage (A stage), those with mild chronic inflammatory cell inflammation as intermediate stage (I stage), and those with no evidence of chronic inflammatory cell infiltration as healing stage (H stage). The process of glandular restructuring was assessed primarily on the basis of glandular density and maturity. Glandular density was classified as low, intermediate, or high, as compared with the lamina propria in the same biopsy specimen. Low was defined as less than half of the glandular density of the normal mucosa, and high as glandular density comparable to that of the normal mucosa. Glandular density between these two grades as classified as intermediate. Glandular maturity was evaluated on the basis of the maturity of cells comprising the glands, and was termed as high, intermediate, or low on the basis of the nucleus : cytoplasm ratio, basophilicity of the cell body for absorptive cells and argyrophil cells; and the number of goblet cells (a remarkable decrease occurs in the active stage). Since the biopsy specimens included from several to more than 10 glands, after evaluating each gland, the overall glandular maturity of the biopsy specimen was evaluated. Overall glandular maturity was evaluated as low when at least half of the glands were immature and high when virtually all of the glands showed normal maturity. Overall glandular maturity between these two grades was evaluated as intermediate.

The Kruskal–Wallis and Mann–Whitney tests were used for statistical analysis. A $P$ value of less than 0.05 was considered to indicate statistical significance.

### 3 RESULTS

### 3.1 Resolution of Inflammation and Colonoscopic Findings

Neutrophil infiltration was contrasted with the stage of inflammation. Among 26 A-stage specimens, 18 (69.2%) showed neutrophil infiltration. Of 20 I-stage specimens 4 (20.0%) showed neutrophil infiltration. The incidence of neutrophil infiltration was significantly higher in A stage than in I stage. In contrast, there was no significant difference between I stage and H stage (Fig. 4(a)).

#### *3.1.1 Vascular Markings*

Of the 26 A-stage specimens, 23 (88.5%) showed no vascular markings. Among the 20 I-stage specimens, only 5 (25.0%) had no vascular markings. The proportion of specimens with no vascular markings was significantly higher for A stage. No

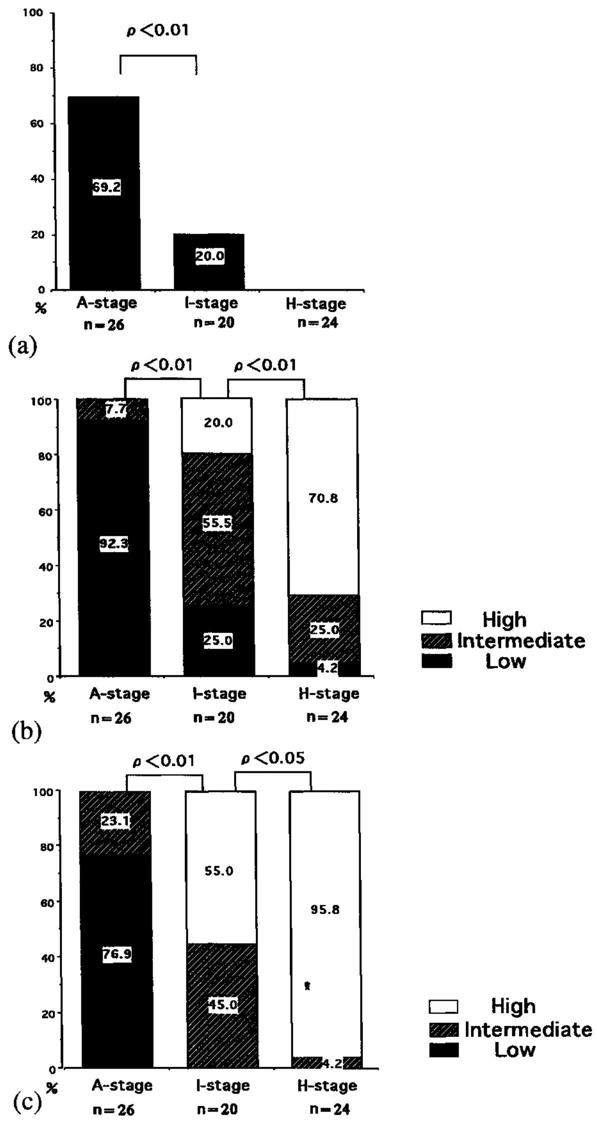

FIGURE 4 Histological findings in each stage. (a) Infiltration of neutrophils; (b) glandular density; (c) glandular maturity.

significant difference was found between I stage and H stage (Fig. 5(a)).

#### *3.1.2 Reddening*

Among the 26 A-stage specimens, 18 (69.2%) showed reddening, while only 5 (25.0%) of the 20 I-stage specimens showed spotty or faint reddening. The incidence of reddening was significantly

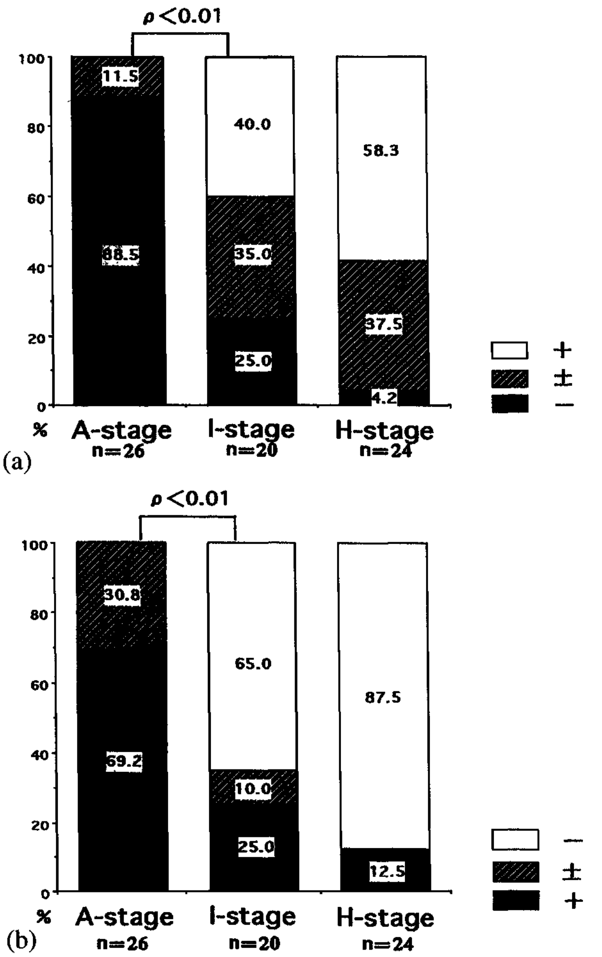

FIGURE 5 Colonoscopic findings in each stage. (a) Vascular markings; (b) reddening.

higher for A stage. There was no significant difference between I stage and H stage (Fig. 5(b)).

### *3.1.3 Erosion and Ulceration*

Among the 26 A-stage lesions, 7 (26.9%) showed erosion and ulceration of 5 mm or more in diameter and 10 (38.5%) showed erosion and ulceration of less than 5 mm in diameter. Of the 20 I-stage lesions, 2 (10.0%) had erosion and ulceration of 5 mm or more in diameter and 1 had erosion and ulceration of less than 5 mm in diameter. The rate of erosion and ulceration was significantly higher in A stage than in I stage. There was no significant difference between I stage and H stage (Fig. 6(a)).

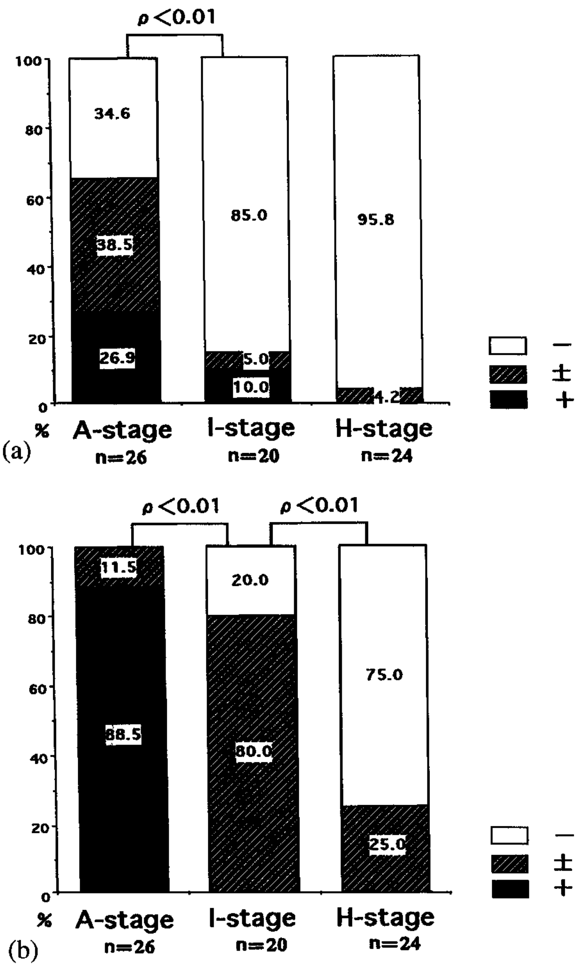

FIGURE 6 Colonoscopic findings after methylene blue dyeing in each stage. (a) Erosion and ulceration; (b) widened grooves.

### *3.1.4 Groove Width*

Of the 26 A-stage specimens, 23 (88.5%) showed consistent evidence of widened grooves. Among the 20 I-stage lesions, no specimen showed consistent evidence of widened grooves. The proportion of specimens with widened grooves was significantly higher in A stage than in I stage. Marginal signs of widened grooves were shown by 16 (80.0%) of the 20 I-stage lesions, as compared with 6 (25.0%) of the 24 H-stage lesions. The rate of specimens with groove widening was significantly higher in A stage (Fig. 6(b)).

### 3.2 Glandular Density and Colonoscopic Findings

When glandular density was classified according to disease stage, A stage had a very high proportion of specimens (92.3%) with low glandular density. In I stage, the majority of specimens (55.5%) showed intermediate density. Among H-stage specimens, 70.8% had high density. The differences among the three groups were significant (Fig. 4(b)).

### *3.2.1 Vascular Markings*

Among the 30 specimens with low glandular density, 22 (73.3%) had no vascular markings. Of the 19 specimens with intermediate glandular density, 4 (21.1%) had no vascular markings. The incidence of no vascular markings was significantly higher among specimens with low glandular density. There was no significant difference in vascular markings between specimens with intermediate glandular density and those with high glandular density (Fig. 7(a)).

### *3.2.2 Reddening*

Of the 30 specimens with low glandular density, reddening was marked in 17 specimens (56.7%) and spotty or faint in 8 specimens (26.6%). Among the 19 specimens with intermediate glandular density, reddening was marked in 4 specimens (21.1%) and spotty or faint in 2 specimens (10.5%). There was a significant difference in the pattern of reddening between specimens with low glandular density and those with intermediate glandular density. No significant difference was found between specimens showing intermediate glandular density and those showing high glandular density (Fig. 7(b)).

### *3.2.3 Erosion and Ulceration*

There was no significant difference in erosion and ulceration of the mucosa among specimens with low, intermediate, and high glandular density (Fig. 8(a)).

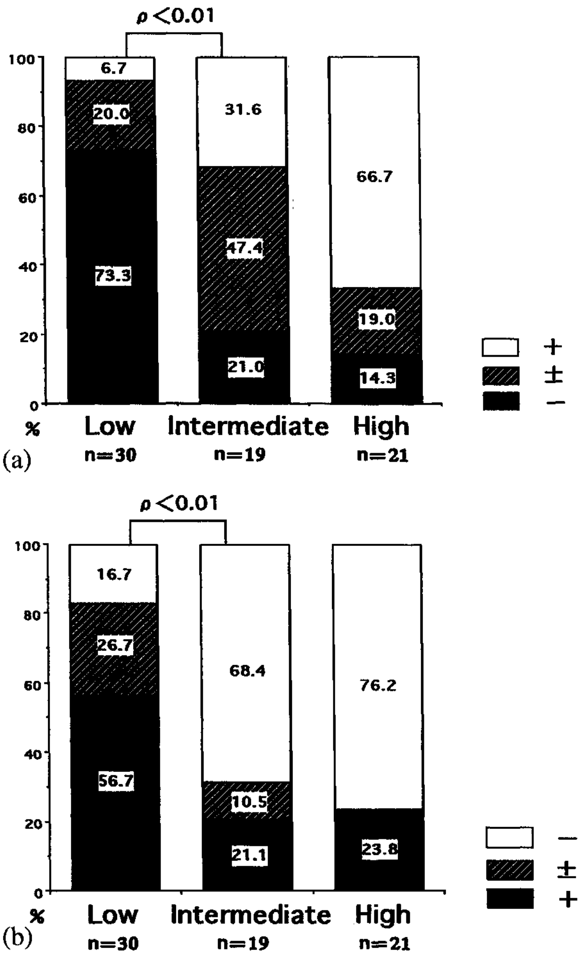

FIGURE 7 Colonoscopic findings according to grade of glandular density. (a) Vascular markings; (b) reddening.

### *3.2.4 Widened Grooves*

Among the 30 specimens with low glandular density, 22 (73.4%) showed consistent widening of the innominate grooves. Of the 19 specimens with intermediate glandular density, only 1 (5.3%) showed widened grooves. The proportion of specimens with consistent evidence of widening grooves was significantly higher for specimens with low glandular density. In addition, among the 19 specimens with intermediate glandular density, 1 (5.3%) had widened grooves (already mentioned), 15 (78.9%), a combination of widened and normal grooves, and 3 (15.8%), normal grooves.

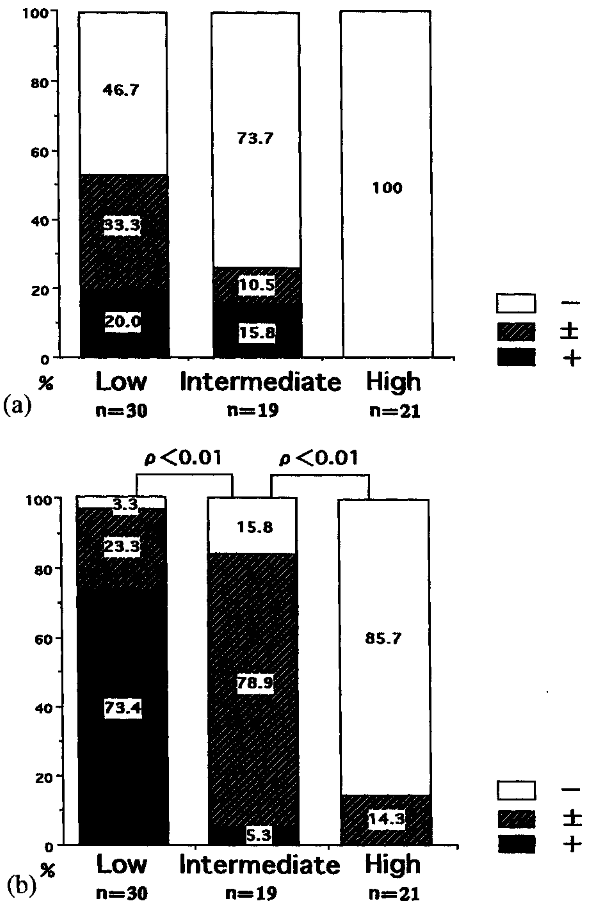

FIGURE 8 Colonoscopic findings after methylene blue dyeing in each grading of glandular density. (a) Erosion and ulcer; (b) widened grooves.

Of the 21 specimens with high glandular density, none showed consistent evidence of widened grooves, 3 (14.3%) showed a combination of widened and normal grooves, and 18 (85.7%) showed normal grooves. There was a significant difference in the incidence of widened grooves between specimens with intermediate glandular density and those with high glandular density (Fig. 8(b)).

### 3.3 Glandular Maturity and Colonoscopic Findings

Glandular maturity was contrasted with the disease stage. In A stage, 76% of specimens had immature glands. In I stage, 45.0% of the specimens showed intermediate gland maturity and 55.0% high gland maturity. In H stage, 95.8% of specimens showed mature glands (Fig. 4(c)).

### *3.3.1 Vascular Markings*

No vascular markings were seen in 20 of the 21 specimens (95.2%) with low glandular maturity and 6 of the 16 specimens (37.5%) with intermediate glandular maturity. The incidence of no vascular markings was significantly higher among specimens with low glandular maturity. Among the 16 specimens with moderate glandular maturity, vascular markings were absent in 6 (37.5%), partly indistinct in 8 (50.0%), and distinct in 2 (12.5%). Among the 33 specimens with high glandular maturity, vascular markings were absent in 3 (9.1%), partly indistinct in 11 (33.3%), and distinct in 19 (57.6%). Specimens with intermediate glandular maturity thus differed significantly from those with high glandular maturity with respect to vascular markings (Fig. 9(a)).

### *3.3.2 Reddening*

Reddening was marked among 15 of 21 specimens (71.4%) with low glandular maturity and 5 of 16 specimens (31.2%) with intermediate glandular maturity. The incidence of marked reddening was significantly higher among specimens with low glandular maturity. Reddening was absent in 6 of 16 specimens (37.5%) with intermediate glandular maturity and 27 of 33 specimens (81.8%) with high glandular maturity. The frequency of no reddening was significantly higher among specimens with high glandular maturity (Fig. 9(b)).

### *3.3.3 Erosion and Ulceration*

Among the 21 specimens with low glandular maturity, the diameter of erosion and ulceration was 5 mm or more in 6 specimens (28.6%) and less than 5 mm in 8 specimens (38.1%). Of the 16 specimens with intermediate glandular maturity,

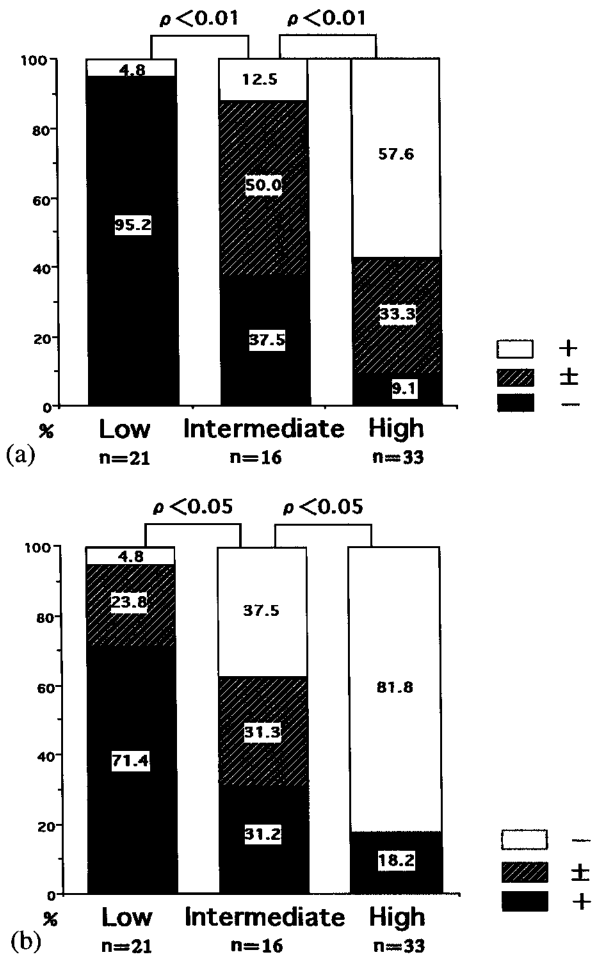

FIGURE 9 Colonoscopic findings according to grade of glandular maturity. (a) Vascular markings; (b) reddening.

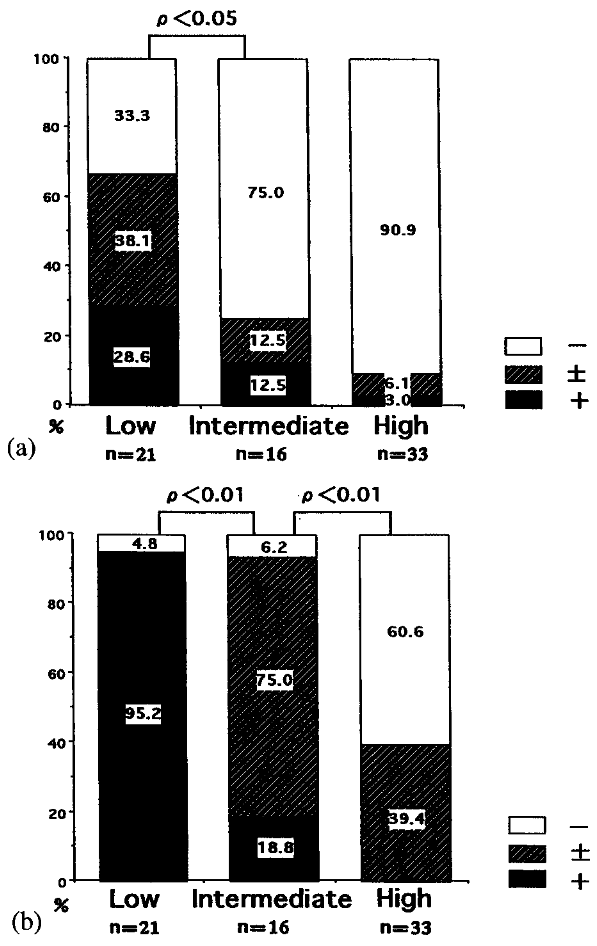

FIGURE 10 Colonoscopic findings after methylene blue dyeing according to grade of glandular maturity. (a) Erosion and ulceration; (b) widened grooves.

the diameter of erosion and ulceration was 5 mm or more in 2 specimens (12.5%) and less than 5 mm in 2 specimens (12.5%). Specimens with low glandular maturity had a significantly higher rate of erosion and ulceration exceeding 5 mm in diameter. There was no difference in erosion and ulceration between specimens with intermediate glandular maturity and those with high glandular maturity (Fig. 10(a)).

### *3.3.4 Widened Grooves*

Consistent evidence of widened grooves was found in 20 of 21 specimens (95.2%) with low glandular maturity and 3 of 16 specimens (18.8%) with intermediate glandular maturity. The incidence of widened grooves was significantly higher for specimens with low glandular maturity. Among the 16 specimens with intermediate glandular maturity, there were widened grooves in 3 specimens (18.8%) and a combination of widened and normal grooves in 12 specimens (75.0%). Of 33 specimens with high glandular maturity, none showed widened grooves, 13 (39.4%) showed a combination of widened and normal grooves, and 20 (60.6%) showed normal grooves. There was a significant difference in the incidence of widened grooves between specimens with intermediate glandular maturity and those with high glandular maturity (Fig. 10(b)).

### 4 DISCUSSION

Various classification systems have been proposed for the active phase of ulcerative colitis, as assessed by conventional endoscopy. These include the classification of Matts [2], modifications of this classification [3,4], the stage-based classifications of Roth [5] and Nagasako *et al.* [6], and the severity-based classifications proposed by Goligher *et al.* [7] and Truelove and Witts [8]. However, endoscopic evidence of the resolving phase, such as the reappearance of vascular markings of the mucosa, may be associated with inflammatory cell infiltration on microscopical examination of a biopsy specimen, and many studies have reported discrepancies between endoscopic and histologic findings [9–12].

Recent progress in endoscopic equipment, especially the availability of endoscopes with magnifying capability, has improved our understanding of early microstructural changes of the mucosa occurring in ulcerative colitis, such as small yellowish spots [3], and areas of gland tubules [4]. In addition, the application of methylene blue dye during endoscopy has facilitated detailed examination of the groove-like pattern of the colon and when combined with magnification, the detection of microstructural changes [13]. Tada *et al.* [14] and Yamakado *et al.* [15] have compared dyeing techniques with conventional endoscopic examination with respect to pathologic and clinical findings in different areas and reported that dye application led to closer correlation between histologic and endoscopic findings. Iso *et al.* [4] studied non-staining areas (areas of gland tubules) by magnifying endoscopy with dye application and imaging analysis and found a close correlation with histologic findings, similar to results in different areas of the colon.

In the present study, we classified cases of ulcerative colitis into disease stage on the basis of not only inflammatory cell infiltration but also characteristics of glandular restructuring (glandular density and glandular maturity) and compared histologic findings with findings obtained by conventional colonoscopy and colonoscopy after dye application.

### 4.1 Evaluation of Resolution of Inflammation and Colonoscopic Findings

Specimens with severe chronic inflammatory cell infiltration (A stage) had a high rate of neutrophil infiltration. Since this rate significantly higher than that among specimens with moderate chronic inflammatory cell infiltration (i.e., I stage) (Fig. 4(a)), the presence of severe chronic inflammatory cell infiltration was associated with the active phase of disease. Neutrophil infiltration was also seen in 20.0% of specimens with mild chronic inflammatory cell infiltration (I stage), but this finding was attributed to factors such as infection or delayed resolution of neutrophil infiltration during the recovery process.

On conventional colonoscopic examination, A stage and I stage differed significantly with regard to vascular markings, reddening, and erosion and ulceration. The absence of vascular markings and reddening appears to indicate the active phase of colitis (A stage), whereas I stage or H stage is characterized by at least the partial appearance of vascular markings and absence of reddening. Matts' classification of endoscopic findings [2] defines Grade 1 as normal vascular markings with no reddening, Grade 2 as the presence of mild reddening with no vascular markings, and Grades 3 and 4 as the presence of pronounced reddening. I stage and H stage thus correspond to Grade 1, and the endoscopic findings of A stage correspond to Grades 2 through 4. When the stages of inflammation are contrasted with Matts' biopsy histologic classification [2], I stage is equivalent to Grade 2. In Matts' classification [2,4], endoscopic findings are evaluated to be milder than the underlying histologic findings. Muto [16] also compared endoscopic and histologic findings and found a concordance rate of 73%, but likewise found that endoscopic findings were milder than histologic changes.

On colonoscopic examination after the application of dye, the rate of ulceration and erosion was found to be higher in A stage than in I stage, indicating that, active disease was accompanied by a high likelihood of some type of tissue damage. Grade 4 of Matts' endoscopic classification [2] includes the presence of ulceration, which corresponds to A stage, but does not include findings obtained after dye application. A direct comparison with our results is therefore inappropriate.

The characteristics of widened grooves on magnified examination after dye application differed among the three stages of disease. Tada *et al.* [14] and Yamakado *et al.* [15] classified specimens according to the shape, arrangement, and demarcation of mucosal grooves between different areas and compared the endoscopic stage classification, as assessed by conventional endoscopy, with the histologic stage classification. They found that the classification of widened grooves showed better agreement with histologic stage than did conventional observations. Our results are consistent with these findings. However, Tada *et al.* [14] and Yamakado *et al.* [15] did not use groove width as a variable, but instead classified grooves into four groups based on size, shape, and margination. Irregular shapes and margins are changes associated with the widening of grooves, and different groove sizes are signs of recovery. Widened grooves alone, as recommended by the author, is both effective and simple. Cole [17] reported that grooves (innominate grooves on barium enema examination) are formed because longitudinal contractions of the muscularis mucosae are disturbed by lymph follicles at the border between the mucosa and the submucosa. When diffuse inflammation develops in the mucosa, the lymph follicles apparently enlarge, and thin grooves in the affected mucosa become shallower and their width becomes broader. Therefore, widened grooves reflect the status of inflammatory cell infiltration and are more useful than other endoscopic findings.

### 4.2 Resolution of Glandular Density and Endoscopic Findings

When contrasted with the stage of inflammation, A stage, I stage, and H stage were characterized by low, medium, and high glandular density, respectively, and the differences among the stages were significant. A stage, or the active stage of disease, is associated with the intercellular infiltration of polymorphonuclear leukocytes, decreased intercellular junctions, and cellular infiltration in the lamina propia of the mucosa [12]. During the process of resolution, inflammatory cell infiltration resolves, and the glandular density is restored. However, almost 30% of specimens had intermediate or low glandular density in H stage, suggesting that decreased glandular density persisted in some cases even after the resolution of inflammation. A chronic decrease in the glandular density has also be reported by Nagasako *et al.* [11], while Nakamura and Kino [18] have found a decrease in glandular density and a relative increase in the thickness of the lamina propia during the remission stage. The biopsy classification of Morson and Dawson [19] characterizes the resolving phase by the presence of epithelial regeneration and restoration of intercellular junctions in the epithelium. Low glandular density in our classification therefore corresponds to the active phase, intermediate density to the resolving phase, and high density to colitis in remission, according to Morson's classification.

### 4.3 Resolution Glandular Maturity and Endoscopic Findings

Morson's biopsy classification [19] proposes that the active phase is associated with the disappearance of goblet cells, which indicates low maturity, whereas the resolving phase is associated with the appearance of goblet cells, which indicates intermediate maturity. In the remission phase, the number of goblet cells is restored, which indicates high maturity. When contrasted with the stage of inflammation, the A stage had the highest rate of specimens with low glandular maturity, while in the I stage 55.0% had high maturity, and in the H stage virtually all specimens had high glandular maturity. These findings indicate that glandular maturity recovers sooner than the resolution of inflammatory cell infiltration or the recovery of glandular density. Inflammatory cell infiltration and glandular maturity therefore appear to be secondary phenomena.

On conventional endoscopy, the three groups of glandular maturity differed significantly with respect to both vascular markings and reddening. Specimens with low glandular maturity had a high rate of no vascular markings, those with intermediate maturity had a high rate of partly indistinct vascular markings, and those with high gland maturity had a high rate of distinct vascular markings. Immature glands were therefore associated with the absence of vascular markings, whereas glands with intermediate maturity had limited vascular markings and mature glands had distinct vascular markings. Next, specimens with immature glands had a high rate of reddening, those with intermediately mature glands had approximately equivalent rates of marked, spotty or faint, and no reddening, and those with mature glands had a high rate of no reddening. Immature glands and some specimens with intermediate glandular maturity thus had intense reddening, whereas mature glands had no reddening.

Therefore, conventional endoscopic findings were more closely correlated with glandular maturity than glandular density of the resolution of inflammation. Conventional endoscopic findings were thus not associated with the resolution of inflammatory cell infiltration (Fig. 4(c)), which requires more time for recovery than glandular maturity. Normalization may occur even when there are remaining signs of inflammatory cell infiltration, and the status of inflammation is evaluated as mild on basis of these conventional endoscopic findings. This may lead to considerable differences between endoscopic findings and histologic findings of biopsy specimens.

Similar to the resolution of inflammation, there were significant differences among specimens with low, intermediate, and high glandular maturity and among the three stages with respect to widened grooves on both staining and magnified examination. Low glandular maturity is a secondary change of inflammatory cell infiltration [12], and widened grooves, which more closely reflect the intensity of inflammation, are apparently a change associated with the degree of glandular maturity.

In conclusion, findings on conventional colonoscopy correlated significantly with the A stage and I stage of the resolution of inflammation, low and intermediate glandular density, and low and intermediate glandular maturity, indicating that the presence or absence of inflammatory activity can be evaluated on the basis of colonoscopic findings. However, there was no correlation of colonoscopic findings with I stage and H stage of the resolution of inflammation or with intermediate and high glandular density; the only correlation was with intermediate and high glandular maturity. It is therefore difficult to evaluate resolution of inflammation (i.e., remission) on the basis of colonoscopic findings. Glandular maturity most closely correlated with conventional colonoscopic findings. Therefore, inflammatory cell inflammation, which resolves after glandular maturity, tended to be underevaluated on the basis of conventional colonoscopic findings. This may account for the differences between endoscopic diagnosis and histologic findings. On magnification after dye application, widened grooves most closely correlated with the resolution of inflammatory cell infiltration from the active to the remission phase. The use of dye application and magnification in conjunction with conventional colonoscopic examination will therefore improve the accuracy of endoscopic diagnosis of the resolution of inflammation to more closely approximate histopathologic findings.

### *Acknowledgments*

I am grateful to Prof. Tetsu Yamaguchi, Prof. Yoshihiro Sakai, and Dr. Sumio Fujinuma,

Lecturer, Third Department of Internal Medicine, Toho University, for their guidance in this study and review of the manuscript. I also sincerely appreciate the help and cooperation of colleagues in the Third Department of Internal Medicine, Toho University.

### *References*

[1] Davis, G.R., Santa Ara, C.A., Morawski, S.G. and Fordtran, J.S. Development of a lavage solution associated with minimal water and electrolyte absorption or secretion, *Gastroenterology* 1980; **78**: 991–995.

[2] Matts, S.G.F. The value of rectal biopsy in the diagnosis of ulcerative colitis. *Quart. J. Med.* 1961; **120**: 393–400.

[3] Saitou, Y. Study on detailed mucosal change of ulcerative colitis comparing pathohistology and mucohistochemistry. *Gastroenterological Endoscopy* 1994; **36**: 263–269.

[4] Iso, A., Fujita, K., Kanda, S. *et al.* Staging of ulcerative colitis by computer analysis of magnifying endoscopic image. *Gastroenterological Endoscopy* 1993; **35**: 474–480.

[5] Roth, J.L.A. *Gastroenterology*, Bockus, H.L. Ed., Second Edn. Vol. II, Philadelphia and London: W.B. Saunders, 1966.

[6] Nagasako, H., Takemoto, C., Ikuzawa, H. *et al.* Endoscopic pictures of the colon (2) ulcerative colitis. *Stomach and Intestine* 1972; **12**: 1671–1678.

[7] Goligher, J.C., de Dombal, F.T., Watts, J.M. *et al. Ulcerative Colitis.* London: Bailliere Tindall and Cassel 1968: pp. 66–102.

[8] Truelove, S.C. and Witts, L.J. Cortisone in ulcerative colitis. *Brit. Med. J.* 1955; **29**: 1041–1048.

[9] Nagasako, K. and Takemoto, T. Endoscopic diagnosis of ulcerative colitis. *Stomach and Intestine* 1976; **11**: 987–996.

[10] Watanabe, A., Hiwatashi, N. and Yamagata, S. Clinical observations on ulcerative colitis. *Japanese Journal of Gastroenterology* 1977; **74**: 57–65.

[11] Nagasako, K., Hasegawa, K., Iizuka, F. *et al.* Evaluation of the role of the biopsy in the diagnosis of ulcerative colitis. *Stomach and Intestine* 1986; **21**: 593–610.

[12] Shiratori, T. *Ulcerative Colitis.* Tokyo: Nankoudou, 1984: pp. 51–61, 154–169.

[13] Sakai, Y., Watanabe, S., Hotchi, Y. *et al.* Detailed diagnosis using dye under colonoscopy. *Endoscopia Digestiva* 1990; **2**: 357–363.

[14] Tada, M., Niki, H., Hattori, S. *et al.* Utility of dye scattering method for the observation of the process of ulcerative colitis. *Gastroenterological Endoscopy* 1975; **17**: 668–674.

[15] Yamakado, S., Sueoka, N., Tamagawa, Y. *et al.* Colonoscopy with an electronic endoscope (TCE50L). *Therapeutic Research* 1987; **6**: 173–180.

[16] Muto, T. *Spectrum of Inflammatory Bowel Disease.* Tokyo: Igakus-Shoin, 1986: pp. 8–36.

[17] Cole, F.M. Innominate grooves of the colon. Morphological characteristics and etiologic mechanisms. *Radiology* 1978; **128**: 41–43.

[18] Nakamura, K. and Kino, I. *Histopathology of Gastrointestinal Tract.* Tokyo: Igaku-Shoin, 1980: pp. 214–226.

[19] Morson, B.C. and Dawson, I.M.P. *Gastrointestinal Pathology.* Second Edn., Blackwell Scientific Publications, Oxford 1979: pp. 293–312, 523–561.

[20] Ajioka, Y., Watanabe, H., Ohta, T. *et al.* Pathological characteristics of colonic erosion. *Endoscopia Digestiva* 1991; **3**: 143–150.

